# Review of health-related quality of life data in multiple myeloma patients treated with novel agents

**DOI:** 10.1038/leu.2013.185

**Published:** 2013-07-16

**Authors:** P Sonneveld, S G Verelst, P Lewis, V Gray-Schopfer, A Hutchings, A Nixon, M T Petrucci

**Affiliations:** 1 Department of Hematology, Erasmus University Medical Center Rotterdam, Rotterdam, The Netherlands; 2 Celgene GmbH, Munich, Germany; 3 OmniScience SA, Geneva, Switzerland; 4 Global Market Access Solutions, London, UK; 5 Oxford Outcomes, an ICON plc. company, Oxford, UK; 6 Sapienza University of Rome, Rome, Italy

**Keywords:** multiple myeloma, quality of life, thalidomide, bortezomib, lenalidomide

## Abstract

In multiple myeloma (MM), health-related quality of life (HRQoL) data is becoming increasingly important, owing to improved survival outcomes and the impact of treatment-related toxicity on HRQoL. Researchers are more frequently including HRQoL assessments in clinical trials, but analysis and reporting of this data has not been consistent. A systematic literature review assessed the effect of novel agents (thalidomide, bortezomib and lenalidomide) on HRQoL in MM patients, and evaluated the subsequent reporting of these HRQoL results. A relatively small body of literature addresses HRQoL data in MM patients treated with novel MM therapeutic agents: 9 manuscripts and 15 conference proceedings. The literature demonstrates the complementary value of HRQoL when assessing clinical response, progression, overall survival and toxicity. However, weaknesses and inconsistencies in analysis and presentation of HRQoL data were observed, often complicating interpretation of the impact of treatment on HRQoL in MM. Further evaluation of HRQoL in MM patients treated with novel agents is required in larger cohorts, and ideally in head-to-head comparative studies. Additionally, the development of standardised MM-specific best practice guidelines in HRQoL data collection and analysis is recommended. These would ensure that future data are more useful in guiding predictive models and clinical decisions.

## Introduction

Multiple myeloma (MM), a clonal proliferation of plasma cells, is an incurable disease in which patients often have pronounced symptoms and substantially reduced health-related quality of life (HRQoL).^1^ Eighty percent of patients experience skeletal destruction,^2, 3^ ∼73% will have anaemia at diagnosis^4^ and ∼30% of patients present with renal insufficiency.^5^ Impaired immune function is also an important characteristic of the disease that leads to severe infections.^6, 7^ Treatment of MM has improved substantially in recent years, leading to prolonged overall survival upon introduction of high-dose chemotherapy combined with autologous stem cell transplantation (ASCT) and use of agents such as thalidomide, bortezomib and lenalidomide.^8, 9^ Despite therapeutic advances, survival prognosis remains poor, with a 5-year relative survival rate of 35–37% in newly diagnosed multiple myeloma (NDMM) patients, although substantial improvements have been observed in patients up to 59 years of age. Survival rates and prognoses decrease with patient age (⩾70 years).^10, 11, 12, 13^ Bortezomib monotherapy and lenalidomide in combination with dexamethasone are both licensed for the treatment of MM patients who have undergone at least one prior therapy. Both therapies have shown statistically and clinically significant improvements over dexamethasone alone in terms of response rates, time to progression and overall survival.^14, 15, 16, 17, 18^ High-dose chemotherapy with ASCT is the standard of care for transplant-eligible, NDMM patients, typically those younger than 65 years of age.^19, 20^ Novel agents, such as thalidomide, lenalidomide and bortezomib, figure prominently in the treatment of these NDMM patients, and are being used in combination with vincristine, adriamycin and/or dexamethasone/low-dose dexamethasone.^21, 22, 23^ These novel agents have also shown efficacy as maintenance treatment post-ASCT, although no drug is currently licensed in this indication.^22, 24, 25, 26, 27^

Novel agents have also changed MM management in elderly patients not eligible for ASCT.^28^ Combinations of melphalan, prednisone and thalidomide (MPT)^29, 30, 31^ and of bortezomib, melphalan and prednisone (VMP)^32, 33^ have shown improved progression-free survival and overall survival compared with melphalan and prednisone (MP) alone. Both MPT and VMP combinations are licensed in Europe for treatment of non-ASCT-eligible NDMM patients and are the current standards of care for elderly patients.^20^ Data also support the role of the combination of melphalan, prednisone and lenalidomide followed by lenalidomide maintenance (MPR-R) in the treatment of elderly non-ASCT-eligible patients,^34, 35^ although it is not currently licensed.

While many of these novel agents have demonstrated improved survival rates, they are also associated with some adverse events (AEs), which can impact on patient HRQoL.^36, 37^ In a prospective study of 154 MM patients in the United Kingdom and Germany, the impact of specific MM symptoms and AEs were correlated with HRQoL scores after adjusting for the level of disease severity. While severe bone pain and being severely symptomatic had the most deleterious effect on patient HRQoL, patients who were receiving MM treatment also reported lower HRQoL, related to treatment toxicity.^38^ It is therefore important to fully understand the effects of treatments on patients' HRQoL. The increasing number of trials evaluating HRQoL in haematological malignancies is a testament to the growing importance of patient-reported outcomes.^39, 40, 41, 42^

The aims of MM treatment are to control disease, prolong survival and maximise patient wellbeing. HRQoL instruments can be incorporated into clinical studies in order to get a more comprehensive evaluation of treatment outcomes. However, the value of HRQoL data in guiding clinical practice depends upon the quality and comparability of these data.

Careful consideration should be given to the study design, including HRQoL instrument selection, a statistical analysis plan and the reporting of results.^43, 44^ Indeed, accumulating evidence indicate that published clinical trials assessing HRQoL have failed to meet good scientific standards of reporting, and internationally agreed upon standards have been called for.^45, 46^

HRQoL data are now routinely captured in studies of new treatments for MM. The objective of this publication was to review available HRQoL data for the newer MM treatments and to critically evaluate the standards of HRQoL data collection, analysis and reporting. It is hoped that our review will help guide the standardisation of HRQoL data collection, analysis and reporting, to work towards either internationally agreed upon guidelines or incorporation into future MM studies.

## Materials and methods

A systematic literature review was performed to identify relevant articles pertaining to the treatment-related HRQoL impact of thalidomide, bortezomib or lenalidomide in MM patients. The following electronic bibliographic and treatment guideline databases were searched: EMBASE, PubMed, National Guideline Clearinghouse Cochrane Database of Systematic Reviews, ClinicalTrials.gov and ClinicalStudyResults.org clinical trial registers (January 2000 through 31 December 2012). Supplementary searches included oncology and haematology conference proceedings (European Hematology Association (EHA), American Society of Clinical Oncology (ASCO), American Society of Hematology (ASH) and International Myeloma Working Group (IMW)), grey literature and reference lists of key papers. The search was restricted to documentation published in human subjects. All interventional study designs involving patients with MM were included, with the exception of case studies. Greater emphasis was placed on randomised controlled trial (RCT) data. Review articles were excluded.

The literature search identified 420 potential publications. After the abstracts for each study were reviewed against the inclusion/exclusion criteria by two independent contributors, 9 manuscripts and 15 conference proceedings (7 ASH, 5 EHA, 2 ASCO and 1 IMW) were considered relevant. Reasons for exclusion included insufficient HRQoL data and non-relevant clinical intervention. The relatively limited amount of retrieved literature was anticipated, given that these therapies are fairly recent and are supported by a limited number of clinical trials that have derived HRQoL analysis. The most frequently used HRQoL instruments in the MM studies included in the current review are briefly outlined in Box 1 and Table 1.

### Studies reporting on HRQoL in MM patients treated with thalidomide

Two clinical trials evaluating MPT vs MP,^47, 48, 49^ and one study evaluating dexamethasone combined with thalidomide (TD) or bortezomib (VD)^50^ were retrieved. Key HRQoL results from these trials are summarised in Table 2.

#### HRQoL data from the HOVON49 phase III study

The HOVON49 randomised, multi-centre, open-label, phase III trial compared MP (*n*=168) with MPT (*n*=165) followed by thalidomide maintenance in elderly (>65 years) NDMM patients.^51^ The MPT regimen has become a standard treatment in this population, based on data from five RCTs.^29, 30, 48, 51, 52, 53, 54^ Eight treatment cycles were planned in the HOVON49 study. Patients who completed the planned MPT cycles received thalidomide maintenance therapy until progression, whereas patients in the MP arm received no maintenance. After disease progression (PD) or no response, salvage therapy was given according to the physician's choice. HRQoL was evaluated as a secondary end point in the study using the QLQ-C30 and QLQ-MY24 questionnaires (Table 1). HRQoL was measured at five pre-determined time points during the course of treatment.^47^

Both treatment arms resulted in improved overall Global Health Status (GHS)/Global QoL, Fatigue, Side Effects of Treatment, Pain, Insomnia and Appetite Loss scores, although differences in favour of MPT were observed for these latter three scores. However, MPT was associated with a significant increase in paraesthesia from post-induction onwards, consistent with a cumulative dose-dependent effect of thalidomide.^51, 55^ The higher incidence of constipation and paraesthesia with MPT vs MP was not reflected in overall HRQoL. No unfavourable overall difference in self-reported side effects between the two arms was observed during the study protocol. Verelst *et al.* explored whether the improvement in HRQoL from baseline seen was clinically significant. The MM-specific minimally important difference (MID) for the QLQ-C30 was defined as a difference of 6–17 points, as estimated by Kvam *et al.*^56^ MID is defined as the smallest change in an HRQoL score considered important to patients that would lead the patient or clinician to consider a change in therapy. It was concluded that a clinically significant difference was observed for GHS/QoL, Role Functioning, Emotional Functioning, Social Functioning, Fatigue and Pain at the end of post-induction (18 months, during the period following the first week after induction treatment until the start of the next treatment off protocol) in favour of MPT-treated patients compared with MP. This prospective study showed that the higher frequency of toxicity associated with MPT did not translate into a negative effect on HRQoL and that patients on MPT have a better outlook. The authors concluded that MPT improved clinical outcome with no reduction in HRQoL (Table 2).

#### Critical review of HOVON49 HRQoL data

The use of a prospective design allowed the evaluation of HRQoL at different time points and treatment stages, with repeated measurements, owing to a linear mixed statistical model, which took into account correlation between measurements from the same patients. The model allowed a clear distinction between differences in the two randomisation arms at baseline and those possibly caused by additional thalidomide treatment. Thus, differences present at baseline did not generate significant interaction with time.

The HRQoL questionnaires were first completed by patients before treatment but after randomisation. The HOVON49 study authors hypothesised that anticipation of receiving beneficial treatment may have biased HRQoL reporting. As the study was open-labelled, patients in the MPT arm may score better at baseline. Indeed, at baseline, the MPT cohort had statistically significantly higher HRQoL scores for QLQ-C30 Emotional Functioning and GHS/QoL subscales, and for QLQ-MY24 Future Perspectives and Social Support subscales.

The quality of the data may be diminished by the open-label study design, which risks incorporating bias, and is further limited by the fact that not all HOVON49 trial patients participated in the HRQoL survey. Compliance rates with completing questionnaires at different study time points were not reported. Furthermore, although patients were evaluated at pre-determined treatment time points, not all patients completed questionnaires at the same time. Finally, although clinically meaningful MID thresholds for MM were applied,^56^ within-trial and domain-specific distribution-based MID estimates would have provided additional insight into the clinically meaningful changes.^43^

#### HRQoL data from the Nordic Myeloma Study Group (NMSG)

Waage *et al.* conducted a double-blind, placebo-controlled study in untreated elderly NDMM patients randomised to receive MPT (*n*=182) or MP (*n*=175) (Table 2). HRQoL was assessed as a secondary end point. Generalised estimating equations were applied, making full use of repeated quarterly measures (Table 1) and allowing for within-patient correlations over successive time points. Analyses were carried out using the observed values of QLQ-C30 scores, including baseline (pre-randomisation) QLQ-C30 scores as covariates.^48, 49^

In both treatment arms, HRQoL improved after treatment initiation. Little difference was detected between treatment arms, although significant differences were observed in favour of MP in Physical Functioning (*P*=0.025) and Social Functioning (*P*=0.013). There was a marked increase in the Constipation score among patients in the MPT arm (*P*<0.001), and a corresponding tendency to an increase (HRQoL worsening) in the Diarrhoea score in MP patients (*P*=0.002). Compliance with completing the QLQ-C30 questionnaire was 82% in the MPT arm and 90% in the MP arm at 3 months, and 50% and 62% at 12 months, respectively.

#### Critical review of NMSG HRQoL data

The main advantage of this HRQoL data is the study's double-blind, placebo-controlled design, which eliminates reporting bias. Multiple imputation of missing data values showed no evidence of bias in the comparison of treatments. However, the HRQoL data were substantially weakened in this double-blind trial by poor compliance in questionnaire completion as the study progressed.

#### HRQoL data from the NMSG (Hjorth *et al.*)

Thalidomide- and bortezomib-naive patients with melphalan-refractory myeloma were randomly assigned to low-dose TD (*n*=67) vs VD (*n*=64) in an open phase III randomised multi-centre trial conducted by Hjorth *et al.* HRQoL was assessed as a secondary end point, measured by the QLQ-C30 questionnaire (Tables 1 and 2). The questionnaire was completed by 96% of patients still alive at 6 weeks, 90% at 12 weeks and by 76% patients at 6 months. No HRQoL improvement over time was observed for either treatment group. No between-group differences were noted, except that the Fatigue score was worse at 12 weeks (*P*=0.04) in the VD group. A higher Sleep Disturbances score was also noted in the VD group at 6 (*P*=0.06) and 12 weeks (*P*<0.01), potentially related to neurotoxicity.

#### Critical review of NMSG HRQoL data (Hjorth *et al.*)

The trial was prematurely closed because of low accrual and was therefore weakened by the low number of recruited patients. However, it remains pertinent, as no other randomised data comparing thalidomide with bortezomib are available.

All domains of the QLQ-C30 were collected and reported for all time points, and all patients were included in the analysis in accordance with intention to treat principle. However, there was relatively little description of how HRQoL data were analysed, and it was not possible to infer how missing data were treated.

Clinically meaningful thresholds were used in interpreting HRQoL results, but a single MID of ⩾10 points was used across all domains.^57^ Compliance in questionnaire completion was high. The HRQoL questionnaires were first completed before randomisation, thus eliminating bias in HRQoL reporting at that time point. As in the HOVON49 trial, the quality of the data is diminished by the open-label design.

### Studies reporting on HRQoL in MM patients treated with bortezomib

Three publications reported HRQoL results for bortezomib treatment in the relapsed/refractory multiple myeloma (RRMM) setting,^58, 59, 60^ covering the APEX phase III and SUMMIT phase II trials.^17, 61^ The phase III VISTA trial reported HRQoL results for bortezomib treatment in elderly NDMM patients.^62^ These publications were also discussed in earlier conference proceedings,^63, 64, 65, 66, 67^ which also reported on the UPFRONT clinical trial in NDMM patients.^68, 69, 70, 71^ Key HRQoL results from these trials are summarised in Table 3.

#### HRQoL data from the APEX phase III trial

The APEX study was a randomised, open-label trial comparing bortezomib (*n*=296) with high-dose dexamethasone (*n*=302) in patients with relapsed MM, evaluated with the QLQ-C30 and the FACT-Ntx questionnaires (Table 1). Assessment of HRQoL was included as a pre-specified exploratory efficacy objective. The APEX trial was stopped early owing to a 29% vs 52% progression rate in favour of bortezomib. In the bortezomib arm, 9% completed all protocol-specified treatment, while in the dexamethasone arm, 5% completed treatment.^60^ HRQoL assessments were discontinued when patients stopped protocol treatment, leading to a high amount of missing data.

At baseline, mean QLQ-C30 scores were significantly better for bortezomib vs dexamethasone in Emotional Functioning, Fatigue, Sleep and Diarrhoea. Baseline FACT-Ntx scores were comparable across groups. HRQoL scores during the 42 weeks of the trial were analysed using generalised estimating equation analysis of covariance.^72^ QLQ-C30 analysis found significantly better HRQoL in the bortezomib group vs dexamethasone, although a declining trend in mean GHS score was observed in both arms (Table 3). The component scores for Physical, Role, Cognitive and Emotional Functioning, and the symptom scores for Dyspnoea and Sleep were significantly better for the bortezomib group. For the overall FACT-Ntx score, statistically significant differences favouring the bortezomib arm were reported, when missing data due to patient death were imputed as worst possible score (zero), but this difference became nonsignificant when treated as withdrawals/missing data.

#### Critical review of APEX HRQoL data

The APEX trial was an open-label randomised study. This landmark trial reported an HRQoL benefit of bortezomib over dexamethasone. There were some differences in the two treatment arms at baseline: the bortezomib group reported better functioning and fewer symptoms than the dexamethasone group. These differences may be owing to chance, as the treatment arm was randomly assigned, HRQoL assessments were made before therapy started and the two groups were balanced for clinical characteristics. In responding patients, HRQoL changes from baseline were similar for most domains, except for better sleep and more neurotoxicity in bortezomib-treated patients, and less nausea and anorexia in the high-dose dexamethasone-treated group, consistent with clinical experience. The analysis may have been more robust if the HRQoL changes over time had been taken into account. The number of patients excluded from the HRQoL analyses was disclosed (*n*=45, because a baseline assessment was missing or because only a baseline assessment was completed). The study also acknowledged that missing data increased with time, owing to AEs that led to discontinuation, PD, premature termination of the dexamethasone arm of the study and death. Although the number of patients completing treatment cycles was reported, the study did not specify compliance rates.

The study assessed the change in HRQoL scores over time by comparing the change in scores according to clinical response between baseline and the best clinical response (best endpoint).^60^ The choice of ‘best endpoint' for HRQoL analysis rather than a combination of data points throughout the trial could be considered selective. This approach may introduce bias, as AEs or symptoms occurring at other time points would be discounted.

Furthermore, the statistical analysis plan for the evaluation of the HRQoL data pre-specified four analytical methods, including two that included and two that excluded multiple data imputations, each combined with deaths assigned a zero value or deaths treated as withdrawals. Details on GHS/QoL data were primarily reported based on the method with no multiple data imputation, and deaths assigned a zero value, reporting an HRQoL benefit for bortezomib. No statistically significant differences in GHS/QoL between bortezomib and dexamethasone were observed with multiple data imputations and deaths treated as withdrawals.^60^ The way in which deaths were treated had an important impact on data interpretation. As acknowledged by the authors, this approach likely biased the results in favour of bortezomib, given the significant survival benefit seen in the trial.^16^

#### HRQoL data from the SUMMIT phase II trial

SUMMIT was an open-label, single-arm trial of bortezomib in 202 patients with RRMM after at least two previous treatments. The study design was described previously.^61^ During the first two cycles, all patients received bortezomib, and dexamethasone could be added for patients with stable disease or PD after four cycles. The study reported rates of 96–97% HRQoL completion at baseline and 76–77% at close-out.

The trial analysed HRQoL results (Tables 1 and 3) reported by clinical response in order to predict the prognostic value of HRQoL for survival in MM.^58, 59^

For the total patient population with available clinical response information (*n*=151) and available HRQoL data (*n*=144), there was a positive change between baseline and best end point. Changes in HRQoL scores showed statistically significant differences between response groups with HRQoL improvement in responding patients (with complete response (CR) or partial response), overall stable scores in patients with minor response or stable disease, and overall decreased scores in PD patients.^58^

The value of baseline HRQoL data in predicting mortality during treatment was analysed through univariate and multivariate logistic regression and by partial least squares regression. Fifteen baseline HRQoL parameters were significant in predicting mortality during treatment when univariate logistic regression was used, but only the QLQ-C30 fatigue and physical subscores were significant predictors of survival in a subsequent multivariate regression (Table 3).^59^

#### Critical review of SUMMIT HRQoL data

As a treatment comparison arm was not included, the impact of treatment on HRQoL changes is difficult to interpret. The study did not present the mean HRQoL change from baseline by cycle or domain. The specified analytical end point assessed the change in HRQoL scores over time by comparing the change in scores according to clinical response between baseline and the best clinical response to treatment.^58^ This choice of ‘best end point' for HRQoL analysis rather than pre-determined data points throughout the trial is selective and was not justified in the reporting.

#### HRQoL data from the VISTA phase III trial

The phase III VISTA trial was a randomised, open-label, multi-centre study performed to assess VMP (*n*=344) vs MP (*n*=338) treatment on overall survival and other clinical benefits in elderly patients with previously untreated MM and patients not eligible for SCT.^62, 66, 67^ HRQoL was an exploratory end point of the VISTA trial. Patients were followed up over 54 weeks (nine 6-week cycles) and post treatment. The QLQ-C30 questionnaire was completed at screening, on day 1 of each treatment cycle and every 8 weeks until progression during follow-up (Table 1). A sustained HRQoL improvement was defined as a change in score of at least 5 points for at least two consecutive cycles after best response, as described by Dubois *et al.*^58^ During early treatment cycles, an overall deterioration in HRQoL was observed in VMP patients, both vs baseline and MP-treated patients. At the cycle 4 assessment, mean differences between the VMP and MP arms were deemed clinically meaningful (⩾5 points) and statistically significant (*P*<0.05) for all domain scores, except for Cognitive Functioning, Nausea/Vomiting, and Dyspnoea. From cycle 5 onwards, a general increase in all HRQoL domain scores was reported in VMP-treated patients vs baseline and MP-treated patients (Table 3).^62^

The VISTA trial also evaluated the impact of clinical response on HRQoL across both treatment arms. Mean scores improved overall in responding patients from time of response to end-of-treatment assessment, especially in patients achieving CR. Multivariate analysis showed a significant impact of duration of response/CR on improving GHS/QoL, Pain, Appetite Loss and Diarrhoea scores (*P*≤0.03 for all). Sustained HRQoL improvements of ⩾5 points were seen following achievement of response.^62^

The impact of bortezomib dose on HRQoL was also evaluated in VMP-treated patients at the time of, and one and two cycles before, both best response and the end-of-treatment visit. Patients receiving a lower dose intensity of bortezomib (<5.6 mg/m^2^/cycle) for at least two cycles before achieving overall response or before their end-of-treatment visit generally reported better HRQoL vs the higher dose intensity group.^62^

#### Critical review of VISTA HRQoL data

The open-label VISTA study is to date the largest randomised, multi-centre study in previously untreated, transplant-ineligible MM patients reporting on HRQoL, with HRQoL collected as an exploratory end point. HRQoL analyses were restricted to data collected from baseline to the end-of-treatment visit, owing to low questionnaire completion rates post-treatment. The results, however, demonstrated that HRQoL is not compromised in the long term with VMP vs MP.

While the number of patients for whom HRQoL data were available and comparable across the study, no between-group comparisons of patient and disease characteristics or compliance rates were reported. This is important, given that the authors did no imputation for missing data. Patients may drop out of the study because of poor response or side effects, which may result in overestimations of HRQoL scores.

Additional analysis investigated differences in HRQoL within treatment arms using multivariate linear regression. The improvement in some HRQoL domains identified after ‘best response' may be influenced by the fact that HRQoL scores for VMP patients were lowest at the point of CR, due to toxicity issues.

Exploratory, *post hoc* analyses by dose intensity provide some evidence regarding the impact on HRQoL of bortezomib dose intensity. The VISTA study authors acknowledged that the study was not designed to compare HRQoL during the periods of twice-weekly and once-weekly bortezomib dosing, so the evidence on higher HRQoL with lower dosages of bortezomib is preliminary. Further studies will be required to confirm a statistical relationship between bortezomib dose intensity and HRQoL.

#### HRQoL data from the UPFRONT phase IIIb trial

In an ongoing randomised, open-label, multi-centre clinical trial that compared the efficacy and safety of three bortezomib-based regimens in untreated, transplant-ineligible NDMM patients, Niesvizky *et al.* described HRQoL data as a primary objective from a total of 300 patients (100 patients per arm) who completed the QLQ-C30 questionnaire. The phase IIIb UPFRONT study compared the safety and efficacy of VD with thalidomide and dexamethasone (VTD), and with melphalan and prednisone (VMP), followed by bortezomib maintenance therapy (Table 3).^68, 69, 70, 71^

Scores improved in all three treatment arms, except for Physical Functioning, Role Functioning and GHS/QoL, which worsened in the VTD arm.^68, 69, 70, 71^ The observed data, linear mixed model estimates and sensitivity analyses all showed a common trend to a transient decrease in HRQoL during VD, VTD and VMP induction, followed by a subsequent trend to improvement/stabilisation in HRQoL during single-agent bortezomib maintenance.

A significant worsening (reduction) (*P*<0.05) in mean GHS/QoL score at cycle 7 from baseline was reported in the VTD and VMP arms (linear mixed effect model). A sensitivity analysis used last observation carried forward for patients with missing data, showing a significant worsening from baseline at cycle 7 in all bortezomib-based treatment arms (*P*<0.05). Symptom scores changed very little during induction with all bortezomib-based regimens, with moderate HRQoL improvements seen during maintenance (except for Nausea, Vomiting and Diarrhoea).

Niesvizky *et al.* concluded that the trend to declining HRQoL during induction may reflect the onset of treatment-associated toxicity. Subsequent HRQoL improvement may reflect the positive impact of achieving a response. The transient decline in HRQoL observed in this study is similar to the trend previously reported in the VISTA study.^66^

#### Critical review of UPFRONT HRQoL data

The UPFRONT data were presented in abstract/poster format, which limits the scope for in-depth critical evaluation. The trial design was open-labelled, but given the fact that all patients received the investigational product, the potential for enhanced response in patients who know that they are receiving an investigational therapy can be excluded.

In terms of compliance, HRQoL assessments were available at baseline and at least one post-baseline time point for 80% (VD), 67% (VTD) and 80% (VMP) of patients. The information on compliance rates is fairly unspecific, as it does not provide sufficient information on overall compliance per treatment cycle, and in particular during the maintenance phase.

The UPFRONT study authors concluded that post-induction improvements/stabilisation in HRQoL may reflect the beneficial impact of achieving a response and the limited toxicity profile associated with weekly bortezomib maintenance. However, unhealthier patients may not have completed HRQoL questionnaires at later stages of treatment, for example, owing to neurotoxicity, leading to a potential bias in reporting.

For patients who died within the HRQoL evaluation period, missing HRQoL assessments were assigned a score of zero, representing the worst possible HRQoL score. While potentially exaggerating the HRQoL of treatments that reduce mortality, this aspect is unlikely to have been a major issue in this trial, given the similar survival rates observed.

### Studies reporting on HRQoL in MM patients treated with lenalidomide

Two studies reporting HRQoL data in MM patients treated with lenalidomide were identified in the search, including comparisons of MPR-R vs MPR vs MP in NDMM patients above the age of 65 (MM-015 trial),^73, 74, 75, 76^ and lenalidomide plus high-dose dexamethasone in RRMM patients (MM-018 trial).^77, 78, 79^ Key HRQoL results from these trials are summarised in Table 4.

#### HRQoL data from the MM-015 phase III trial

The MM-015 phase III trial was a multi-centre, randomised, double-blind, placebo-controlled, three-arm parallel-group study to determine the efficacy and safety of MPR-R (*n*=152) vs MPR (*n*=153) and MP (*n*=154) in NDMM subjects who are 65 years of age or older (Table 4). This study investigated HRQoL as a secondary outcome at baseline and the beginning of every third cycle, and at time of progression (PD) or discontinuation for reasons other than progression (DC). HRQoL was assessed using the QLQ-C30 and QLQ-MY20 for the following pre-selected HRQoL domains: GHS/QoL, Physical Functioning, Fatigue and Pain (QLQ-C30), and Side Effects of Treatment and Disease Symptoms (QLQ-MY20) (Table 1).

In all three study arms, HRQoL improvement was observed from baseline to study end for all specified HRQoL domains. Statistically significant HRQoL improvements (*P*<0.05) from baseline to cycle 10 (end of induction phase) were observed in each domain for MPR-treated patients, with the exception of Side Effects of Treatment (nonsignificant for all treatment arms). Improvements in HRQoL in patients aged 65–75 years were comparable, or slightly greater, to those in all patients aged ⩾65 years.

Comparably favourable HRQoL results could not be replicated for the subset of patients aged ⩾75 years, owing to the limited number of patients. Changes in HRQoL from cycle 10 to cycle 16 were not statistically significant (*P*>0.05) in any HRQoL domain, suggesting that the majority of HRQoL improvement occurred during the induction phase and stabilised during maintenance.^76^ With regards to changes from baseline at cycle 16, there were statistically significant changes in the MPR-R group for all domains (again with the exception of Side Effects of Treatment), while statistically significant changes were not consistently observed for MPR and MP patients.^76^

A mixed model repeated measures analysis for the QLQ-C30 and QLQ-MY20 scores indicated a significant difference in changes in Physical Functioning scores across treatment groups: scores improved significantly (*P*<0.05) from baseline in MPR-R and MPR patients but worsened in MP patients.^76^

Comparisons of HRQoL scores using trial- and domain-specific MIDs were also conducted and time points at which mean changes from baseline for each arm exceeded the MID were presented.^75, 76^ In this study, the standard error of measurement was used to establish MID.^80, 81^ The MPR-R group mean change from baseline exceeded the MID more frequently than was observed in the other two treatment groups. MID HRQoL improvements were observed as early as cycle 4 for Pain. MIDs were applied to investigate the percentage of patients who had a change from baseline exceeding the MID at cycle 10 and 16 for MPR-R and MP treatment groups. A higher number of patients in the MPR-R group exceeded the MID for all domains.

An additional analysis presented results from a mixed-effects multiple regression model that estimated which clinical parameters were associated with statistically significant and clinically meaningful improvements in HRQoL in the MPR-R and MP treatment arms. More patients achieved very good partial response or better (⩾VGPR) when receiving continuous MPR-R treatment than those receiving MP. ⩾VGPR was shown to improve GHS/QoL in a clinically meaningful and statistically significant way, suggesting that clinical responses with MPR-R treatment were not related to HRQoL impairments due to treatment-related toxicity. PD was also shown to negatively impact GHS/QoL (−8.34; *P*<0.001), with MPR-R significantly reducing the risk of PD vs MP. Continuous MPR-R may therefore delay PD and help improve and maintain HRQoL.^74^

#### Critical review of MM-015 HRQoL data

The percentage of patients completing questionnaires until cycle 16 was consistently above a threshold of 76%, with compliance rates above 65% at PD/DC. The percentage of compliant subjects was not significantly different between treatment arms at any of the visits, except at cycle 7 for the QLQ-MY20 questionnaire (*P*=0.036).^76^

Mean HRQoL domain scores were presented for each treatment at each measurement time point, along with longitudinal differences between treatment arms.

Significant emphasis in the presentation of the findings, as reflected by most reported comparative analyses between treatment arms, was placed on ‘responder' analyses (percentage of patients achieving a clinically meaningful HRQoL response in each arm), using MID as the definition of clinically meaningful response.

Patients randomised to MPR-R had worse HRQoL scores compared with the other arms, but the difference was statistically significant only for Physical Functioning (*P*=0.014). Sensitivity analyses and inclusion of ‘time × arm' interactions accounted for baseline differences. Mixed models were used to estimate the treatment effect on HRQoL over time, adjusted and unadjusted for baseline HRQoL scores.

The study used repeated measures mixed-effects modelling to account for missing variables and described the extent of missing data overall and by treatment arm. There were no statistically significant differences in demographics and disease-related characteristics between the three treatment arms at baseline, cycle 10 or cycle 16, suggesting that there was no significant difference between treatment arms in patients who dropped out or were non-compliant.

#### HRQoL data from the MM-018 phase III trial

In the MM-018 phase III single-arm, open-label study, lenalidomide plus high-dose dexamethasone was administered to 587 RRMM patients in the United Kingdom, Spain and Ireland, to assess the safety of this regimen and its impact on HRQoL (Table 4). Secondary outcome HRQoL assessments were conducted at baseline and after 24 weeks of treatment using the QLQ-C30 and QLQ-MY20 questionnaires (Table 1).

QLQ-C30 revealed no significant median change (>5 points MID) from baseline in 14 of 15 domains for patients completing questionnaires at baseline and 24 weeks. Median Fatigue increased in the United Kingdom/Ireland population (score 11.1). QLQ-MY20 revealed no significant median change from baseline of all scores except an improvement in Future Perspective in Spanish patients (median 11.1), for patients completing questionnaires at baseline and 24 weeks.^79^ Alegre *et al.*^77, 78^ reported further HRQoL data from 63 patients enrolled in the Spanish cohort. At week 24, 42 patients were available for HRQoL assessment. In addition to the reported improvement in Future Perspective, a nonsignificant impairment in the Physical Functioning domain of the QLQ-C30 functional scores was also observed (<5 points MID). The majority of patients who experienced HRQoL changes according to QLQ-C30 and QLQ-MY20 scores had clinically meaningful improvements in HRQoL, regardless of response (20/42 patients achieved either a CR or VGPR during treatment). Despite comedication with high-dose dexamethasone, pre-existing neuropathy in >50% patients, prior MM treatment, and late disease course, patients were able to maintain median QoL scores over 24 weeks.^77, 78, 79^

#### Critical review of MM-018 HRQoL data

The open-label, single-arm study design did not allow for reliable inference on the extent to which treatment truly impacts upon HRQoL. The data were collected at two end points (baseline and week 24) and are therefore likely to be incomplete for some patients (for example, 42/63 patients completed HRQoL at 24 weeks in the Spanish subset). Unlike the APEX trial for bortezomib, no information on missing data or withdrawals was reported. This may constitute a major weakness especially among an RRMM population where missing data are more likely to introduce potential bias.

## Conclusions

To date, there has been a relatively small body of HRQoL data published on novel MM treatments. Available HRQoL data do not allow for comparisons of HRQoL impact across MM treatments, owing to differences in patient population, lack of comparative trials, differences in study designs and in methodology applied for the specific HRQoL analysis.

Patient groups differ between trials, for example, in terms of age distribution and pathology (NDMM or RRMM) being particularly diverse, which may impact on HRQoL. Baseline HRQoL values were significantly different between treatment groups in the thalidomide HOVON49 and the bortezomib APEX trials. In cases where baseline HRQoL results are statistically significantly different between treatment arms, it is important to conduct sensitivity analyses in order to control for baseline differences in HRQoL, as was done in the MM-015 trial.

Study design may also impact on interpretation of HRQoL outcomes. Few studies were double-blind RCTs, such as the lenalidomide MM-015 and thalidomide Waage *et al.* trials, making inference for all other trials more difficult. Unblinded studies (for example, the thalidomide HOVON49 and all retrieved bortezomib trials) may increase the potential for an enhanced response in patients who are aware they are receiving an investigational treatment. Even though a number of studies were open-labelled (for example, thalidomide HOVON49 and Hjorth *et al.* trials, and bortezomib APEX, VISTA and UPFRONT trials), their value in terms of inference is superior to single-arm trials (for example, bortezomib SUMMIT and lenalidomide MM-018 trials). Furthermore, some studies have only been reported as conference proceedings (UPFRONT and extended analyses on clinical parameters affecting HRQoL in MM-015).

Differences in interpreting clinically meaningful MID thresholds for MM were observed across retrieved studies. However, there is no unanimous agreement on what constitutes clinically meaningful MID changes per HRQoL domain, allowing for several different definitions of MIDs as well as methods of analysis.

All retrieved studies explored HRQoL as pre-specified secondary end points, except for the bortezomib UPFRONT study, in which measurement of HRQoL changes is described as a primary objective. RCTs measuring HRQoL as a primary outcome have been shown to display higher concordance on pre-specified quality measures.^82^ Consistent use of the well-validated QLQ-C30 and QLQ-MY20 questionnaires facilitates comparisons between treatments. Of note, for individual treatment combinations containing novel therapies, patterns in longitudinal HRQoL trends are generally consistent across the majority of HRQoL domains analysed. There are similarities between studies, regarding timing of HRQoL assessments (Table 1), with common time points at the beginning of treatment cycles. There have, however, been inconsistent approaches to the analysis of HRQoL data across studies in MM, and not all studies have reported the observed HRQoL at each time point for all arms in the study, which would constitute the most straightforward and transparent way to present findings.

Differences in dealing with and reporting missing data were observed across the retrieved studies. The thalidomide trials retrieved in this analysis incorporated all data points at which HRQoL was assessed and presented the observed results at all time points. APEX and UPFRONT trials applied missing data imputations but did not present these findings in detail. In contrast, missing data imputations were not carried out in the thalidomide Hjorth *et al.* trial, in the VISTA and SUMMIT trials for bortezomib, or the MM-015 and MM-018 trials for lenalidomide. However, results from MM-015 were strengthened through mixed model repeated measures analyses. Mixed model repeated measures analyses across time points and paired analyses of data both between treatment arms at individual time points and longitudinally within treatment arms across two time points are methods of assuring that observed changes are not attributable to the changing nature of the sample across time.

Differences in compliance rates between treatment arms, an important pre-requisite for cross-sectional HRQoL data comparisons, were reported in the thalidomide Waage *et al.* and in the lenalidomide MM-015 studies, but were not discussed in the other trials.

Finally, in HRQoL analyses such as APEX, which assume a zero HRQoL score for patients who have died, results may have favoured bortezomib owing to the significant survival benefit established in the study.

As some treatment options have prolonged survival in MM patients, and owing to the impact of treatment-related toxicity on HRQoL, HRQoL data have become increasingly relevant key performance indicators. In the absence of differences in treatment efficacy, the choice of initial treatment should be based on HRQoL, among other patient-related factors. Quality-adjusted survival analyses that integrate HRQoL considerations may be important, particularly in treatments that do not show significant survival advantages.^83^ Guidelines for best practice in collecting and analysing HRQoL in MM would ensure that future data are more useful in informing clinical decisions, whereby more consistent reporting of HRQoL data will improve the understanding of the HRQoL impact of different MM treatments. For those assessing HRQoL in MM studies, our review provides guidance on good practices and standardisation for HRQoL data collection, analysis and reporting (Table 5). The proposed incorporation of HRQoL as a clinically relevant end point in MM drug registration dossiers and in RCTs stresses the need for validated instruments and specific questionnaires, for instance to measure the impact of toxicities such as peripheral neuropathy.^37, 84^ Future HRQoL investigations in MM patients would gain value if head-to-head comparative studies were carried out.

## Figures and Tables

**Table 1:** HRQoL instruments used in MM studies

*Trial (Reference)*	*HRQoL instruments*	*Timing of HRQoL assessments*
*Thalidomide*		
HOVON49^47^	QLQ-C30, QLQ-MY24	Measurements for QoL were collected at study entry, after cycle 3 (∼3 months after the start of cycle 1), after cycle 8 (∼9 months after the start of cycle 1), at 12 and 18 months after the start of cycle 1
Waage *et al.*; Guldbrandsen *et al.*^48, 49^	QLQ-C30	The questionnaires were completed at inclusion and later posted to patients every third month throughout the study
Hjorth *et al.*^50^	QLQ-C30	Questionnaires were completed before randomisation (before start of treatment) and mailed to the patients after 6 and 12 weeks, after 6 months and every 6 months thereafter until the end of the study
		
*Bortezomib*		
APEX^60, 65^	QLQ-C30, FACT-Ntx	Questionnaires were administered at baseline and at weeks 6, 12, 18, 24, 30, 36 and 42
SUMMIT^58, 59, 63, 64^	QLQ-C30, QLQ-MY24, FACIT-Fatigue, FACT-Ntx	Patients completed the questionnaires during screening, on day 1 of cycles 3, 5 and 7 of treatment, as well as at the end of the study
VISTA^62, 66, 67^	QLQ-C30	Patients completed the questionnaire at screening, on day 1 of each cycle during the treatment phase and every 8 weeks until progression during follow-up
UPFRONT^68, 69, 70, 71^	QLQ-C30	Patients completed the questionnaire before dosing on day 1 of cycle 1 (baseline), before dosing on day 1 of every odd-numbered cycle, at the end-of-treatment visit and every 12 weeks thereafter
		
*Lenalidomide*		
MM-015^73, 74, 75, 76^	QLQ-C30, QLQ-MY20	Questionnaires were completed at baseline, at the beginning of every third cycle (cycles 4, 7, 10, 13 and 16), at study discontinuation and every 6 months in long-term follow-up
MM-018^77, 78, 79^	QLQ-C30, QLQ MY20	Questionnaires were completed at baseline and at week 24

Abbreviations: HRQoL, health-related quality of life; MM, multiple myeloma.

**Table 2:** Key results from thalidomide clinical trials reporting HRQoL data

*Trial/references*	*Trial design*	*Key HRQoL results*
*Thalidomide*		
HOVON49^47^	Prospective HRQoL study to assess the impact of thalidomide on HRQoL. Standard MP (*n*=168) vs MPT followed by Thal maintenance (*n*=165) in elderly (>65 years) NDMM patients	Physical Function and Constipation (significant) showed an improvement during induction in favour of the MP arm During Thal maintenance, paraesthesia was significantly higher in the MPT arm, and a trend towards improved Pain, Insomnia, Appetite Loss and the QLQ-MY24 item sick scores was observed The GHS/Global HRQoL scale showed a significant time trend towards more favourable mean values during protocol treatment without differences between MP and MPT For the QLQ-C30 subscales, Emotional Functioning and Future Perspectives, a difference in favour of the MPT arm from the start of treatment was observed, with no significant ‘time × arm' interaction, indicating a persistent better patient perspective with MPT treatment
Waage *et al.*; Guldbrandsen *et al.*^48, 49^	Phase III randomised, double-blind, placebo-controlled study in untreated elderly NDMM patients to compare MPT (*n*=182) vs MP (*n*=175)	Overall, HRQoL outcomes improved equally in both arms, apart from markedly increased Constipation in the MPT arm
Hjorth *et al.*^50^	Open phase III randomised multi-centre trial to compare TD (*n*=67) vs VD (*n*=64) in melphalan-refractory myeloma patients	No differences were noted for Physical Functioning, Pain and GHS/QoL. Fatigue and Sleep Disturbances were more prevalent in the VD group. The Fatigue score for the VD group was worse at 12 weeks, with a score difference of 10 (*P*=0.04). The difference from the time of randomisation in score for Sleep Disturbances in the VD group reached statistical significance at 12 weeks (*P*<0.01)

Abbreviations: GHS, Global Health Status; HRQoL, health-related quality of life; MP, melphalan and prednisone; MPT, melphalan, prednisone and thalidomide; NDMM, newly diagnosed multiple myeloma; Thal, thalidomide; TD, thalidomide and dexamethasone; VD, bortezomib and dexamethasone.

**Table 3:** Key results from bortezomib clinical trials reporting HRQoL data

*Trial/references*	*Trial design*	*Key HRQoL results*
*Bortezomib*		
APEX^60, 65^	Prospective, open-label, randomised, phase III trial of bortezomib (*n*=296) vs Dex (*n*=302) in patients with relapsed MM	Bortezomib was associated with significantly better HRQoL compared with Dex, consistent with better clinical outcomes, although a declining trend in mean GHS score was observed in both arms Patients receiving bortezomib demonstrated significantly better mean GHS over the study compared with patients receiving Dex, as well as significantly better Physical Health, Role, Cognitive and Emotional Functioning scores, lower Dyspnoea and Sleep symptom scores, and better FACT-Ntx questionnaire scores
SUMMIT^58, 59, 63, 64^	Open-label, multi-centre, phase II trial of bortezomib in patients with refractory MM (*n*=202, 193 evaluated)	Change in HRQoL scores showed statistically significant differences between response groups with HRQoL improvement in patients with CR or PR, mostly stable scores in patients with minor response or no change Fifteen HRQoL parameters were significant in predicting mortality when univariate logistic regression was used. When using multivariate regression with stepwise selection to predict survival, only Fatigue and physical subscores were significant predictors of survival
VISTA^62, 66, 67^	Randomised, open-label, multi-centre, phase III study to compare VMP (*n*=344) vs MP (*n*=338) in NDMM patients	Clinically meaningful, transient HRQoL deterioration was observed with VMP vs MP during early treatment (up to cycle 4), followed by HRQoL improvements in VMP-treated patients for all domains, relative to baseline/MP from cycle 5 onwards Overall, mean scores improved in both arms by end-of-treatment vs baseline. Among responding patients, mean scores improved from time of response to end-of-treatment assessment. Multivariate analysis showed a significant impact of response duration or CR on GHS/Global HRQoL, Pain and Appetite Loss. Analyses by bortezomib dose intensity indicated better HRQoL in patients receiving lower dose intensity
UPFRONT^68, 69, 70, 71^	Randomised, open-label, multi-centre phase IIIb trial to compare bortezomib with (i) Dex, (ii) Thal and Dex, or (iii) VMP, followed by bortezomib maintenance in NDMM patients (100 patients per arm)	A trend to decreased HRQoL score was observed in all treatment groups during induction, followed by an increase or stabilisation by the end of treatment There were no differences between treatment arms during induction. Moderate improvements were seen during maintenance, except for Nausea/Vomiting and Diarrhoea

Abbreviations: CR, complete response; Dex, dexamethasone; GHS, Global Health Status; HRQoL, health-related quality of life; MM, multiple myeloma; MP, melphalan and prednisone; NDMM, newly diagnosed multiple myeloma; PR, partial response; Thal, thalidomide; VMP, melphalan, prednisone and bortezomib.

**Table 4:** Key results from lenalidomide clinical trials reporting HRQoL data

*Trial/references*	*Trial design*	*Key HRQoL results*
*Lenalidomide*		
MM-015^73, 74, 75, 76^	Phase III, multi-centre, randomised, double-blind, placebo-controlled, three-arm parallel-group study (MPR-R *n*=152, MPR *n*=153 or MP *n*=154) in NDMM patients ⩾65 years	Clinically meaningful improvements in HRQoL were more frequently observed in patients receiving MPR-R than those receiving MP The differences in HRQoL were most marked in terms of Physical Functioning, and overall results were consistent in patients aged 65 to 75 years A higher percentage of MPR-R patients achieved the MID in six pre-selected HRQoL domains than those receiving MP. Improved or stabilising longitudinal HRQoL trends were observed for most other HRQoL domains
MM-018^77, 78, 79^	Phase III, multi-centre, single-arm, open-label, expanded-access study in RRMM subjects (*N*=587) treated with Len+high-dose Dex in 4-week cycles. Subjects followed until PD or DC	In the Spanish cohort of the study, GHS/Global QoL, Fatigue, Emotional Functioning, Physical Functioning, Role Functioning, Social Functioning, Cognitive Functioning and Pain improved >5 points Preservation of HRQoL correlated with response to treatment in terms of Role Functioning, Emotional Functioning, Social Functioning and Pain scores

Abbreviations: DC, discontinuation; Dex, dexamethasone; HRQoL, health-related quality of life; GHS, Global Health Status; Len, lenalidomide; MID, minimal important difference; MM, multiple myeloma; MP, melphalan and prednisone; MPR(-R), melphalan, prednisone and lenalidomide (and maintenance lenalidomide); NDMM, newly diagnosed multiple myeloma; PD, progressive disease.

**Table 5:** Guidance in collecting and analysing HRQoL in MM patients treated with novel agents based on the current analysis

*Instruments*
• Internationally validated questionnaires, to be used in their entirety
• Questionnaires measuring the impact of treatment toxicity
• Prospective design

*Study design*
• Intention to treat principle
• Preferably randomised double-blind trial
• If study design is a randomised, open-label trial, baseline questionnaire to be completed before randomisation
*Assessment time points*:
• At baseline and at different pre-determined treatment time points
• At the end of treatment, ideally including a differentiation between disease progression and discontinuation
• Regular HRQoL assessments following end of treatment, if possible

*Reporting and analysis*
*Compliance reporting*:
• Compliance rates regarding questionnaire completion at each assessment time point and per study arm
• Statistical between-group comparisons at individual measurement time points, assessing data interpretability independent of absolute compliance levels
• Between-group comparisons of individual patient categories (study drop-outs vs non-compliant vs compliant patients) in terms of patient and disease characteristics and inclusion of treatment interaction terms
*Types of HRQoL assessment*:
• Longitudinal and cross-sectional analysis reporting of HRQoL
• Reporting of individual HRQoL scores at each time point and for each study arm
• Illustration of mean HRQoL changes from baseline over time via repeated measures analysis
• Illustration of both statistical significance and clinical meaningfulness (e.g., with MIDs)
Determination of MID should be based on a combination of statistical reasoning and clinical judgement,^43^ including both trial- and domain-specific analyses
• Linear mixed model per treatment arm across all measurement time points
• Sensitivity analysis controlling for baseline HRQoL and baseline differences in key patient and disease characteristics

Abbreviations: HRQoL, health-related quality of life; MID, minimal important difference; MM, multiple myeloma.

## HRQoL in MM and overview of commonly used HRQoL instruments

• *QLQ-C30*: 30-item, self-administered HRQoL patient questionnaire is designed for use in cancer patients. It has a one-week recall period and is composed of multi-item and single scales, including five functional scales (Physical, Role, Emotional, Social, and Cognitive Functioning), three symptom scales (Fatigue, Nausea/Vomiting and Pain) and a Global Health Status (GHS)/Quality of Life (QoL) scale. Single items include Dyspnoea, Insomnia, Appetite Loss, Constipation, Diarrhoea and Financial Difficulties. The QLQ-C30 has demonstrated good psychometric properties in solid tumours^85, 86^ and reliability and validity in MM patients^87, 88, 89, 90^. The Minimal Important Difference (MID), defined as the smallest change in an HRQoL score considered important to patients that would lead the patient or clinician to consider a change in therapy,^91^ has been estimated for QLQ-C30 in MM patients.^56^

• *QLQ-MY24 and QLQ-MY20*: QLQ-MY24 was developed as an addition to QLQ-C30 for specific use in MM.^92, 93^ The QLQ-MY24 was refined to 20 items (QLQ-MY20). It has a 1-week recall period and addresses four important domains in MM: Disease Symptoms, Side Effects of Treatment, Body Image and Future Perspectives. The psychometrics, including reliability and validity of QLQ-MY20, in MM have been published.^92^

• *QLQ-CIPN20*: the reliability, validity and responsiveness of the 20-item QLQ-CIPN20 instrument for patient-reported chemotherapy-induced peripheral neuropathy (CIPN) is currently being investigated in a wide range of oncology patient populations. The instrument has three subscales: a sensory, motor and autonomic subscale.^94^

• *FACT-Multiple Myeloma*: a 14-item disease-specific FACT-MM HRQoL measure has been developed and has demonstrated acceptable psychometric properties.^95, 96^

• *FACIT-Fatigue*: bolt-on module to the base FACT-General (FACT-G) HRQoL questionnaire, a 27-item instrument measuring Well being (Physical, Social/Family, Emotional and Functional), with a recall period of 1 week.^97^ The MID has been estimated and verified in a group of mixed diagnosis cancer patients.^98^ No reliability, validity or other psychometric properties of the FACIT-Fatigue specific to MM have been described.

• *FACT-NTx*: another FACT-G bolt-on module with additional neurotoxicity parameters. It has demonstrated good psychometric properties in women with ovarian cancer,^99^ but has only been used in a limited number of MM studies, similarly to FACIT-Fatigue.

• *EQ-5D*: standardised generic HRQoL questionnaire that can be converted into a ‘health utility' score questionnaire used to measure health outcomes. The MID has recently been described for EQ-5D in MM patients.^100^

## Notes

Sonneveld P: Advisory Boards and Research support: Janssen, Celgene, Onyx, Millennium; Verelst S: no disclosures; Lewis P: Celgene employee, stock options; Gray-Schopfer V: Consultancy fees as external contractual partner to Global Market Access Solutions (GMAS); Hutchings A: Consultancy fees to GMAS from Celgene; Nixon A: Consultancy fees from Celgene to Oxford Outcomes for MM-015 HRQoL analysis and publications; Petrucci MT Honoraria: Celgene, Janssen-Cilag.
